# Hypermobile Ehlers-Danlos syndrome and spontaneous CSF leaks: the connective tissue conundrum

**DOI:** 10.3389/fneur.2024.1452409

**Published:** 2024-07-17

**Authors:** Sydney Severance, Victoria Daylor, Taylor Petrucci, Cortney Gensemer, Sunil Patel, Russell A. Norris

**Affiliations:** ^1^Department of Regenerative Medicine and Cell Biology, Medical University of South Carolina, Charleston, SC, United States; ^2^Department of Neurosurgery, Medical University of South Carolina, Charleston, SC, United States

**Keywords:** collagen, extracellular matrix, Ehlers-Danlos syndrome, connective tissue disorders, blood–brain barrier, cerebrospinal fluid (CSF) leaks, mast cell activation disorder (MCAD)

## Abstract

Collagen, the most abundant protein in the body, is a key component of the extracellular matrix (ECM), which plays a crucial role in the structure and support of connective tissues. Abnormalities in collagen associated with connective tissue disorders (CTD) can lead to neuroinflammation and weaken the integrity of the blood–brain barrier (BBB), a semi-permeable membrane that separates the brain’s extracellular fluid from the bloodstream. This compromise in the BBB can result from disruptions in ECM components, leading to neuroinflammatory responses, neuronal damage, and increased risks of neurological disorders. These changes impact central nervous system homeostasis and may exacerbate neurological conditions linked to CTD, manifesting as cognitive impairment, sensory disturbances, headaches, sleep issues, and psychiatric symptoms. The Ehlers-Danlos syndromes (EDS) are a group of heritable CTDs that result from varying defects in collagen and the ECM. The most prevalent subtype, hypermobile EDS (hEDS), involves clinical manifestations that include joint hypermobility, skin hyperextensibility, autonomic dysfunction, mast cell activation, chronic pain, as well as neurological manifestations like chronic headaches and cerebrospinal fluid (CSF) leaks. Understanding the connections between collagen, CSF, inflammation, and the BBB could provide insights into neurological diseases associated with connective tissue abnormalities and guide future research.

## 1 Introduction

The central nervous system (CNS) orchestrates the intricate processing of sensory information and motor commands, with its core components—the brain and spinal cord—shielded by the cerebrospinal fluid (CSF) and protected by the blood–brain barrier (BBB). The BBB, a selectively permeable membrane, regulates the passage of substances between the bloodstream and the brain, maintaining CNS homeostasis through its dynamic structure comprising specialized cells and extracellular matrix (ECM) components. Connective tissue disorders (CTDs), such as hypermobile Ehlers-Danlos syndrome (hEDS), pose a challenge to the integrity of the ECM, including collagen, a pivotal protein providing structural support to tissues. Collagen abnormalities in CTDs may compromise the structural and functional integrity of the BBB, contributing to the increased prevalence of CSF leaks and vascular insufficiency observed in hEDS patients. This disruption in BBB integrity may manifest in a spectrum of neurological symptoms, from chronic headaches to cranial nerve dysfunction, underscoring the need for a comprehensive diagnostic approach to identify potential CSF leaks in patients with suspected hEDS.

Understanding the underlying mechanisms linking ECM changes in hEDS to BBB alterations and subsequent CSF leaks is crucial for navigating the treatment of the neurological manifestations of hEDS. Moreover, mast cell activation disorders (MCAD) and consequences from mediator release may be a potential contributor, highlighting the importance of investigating the role of mast cell activity in preventing recurrent leaks. In light of the underdiagnosis of both hEDS and CSF leaks, a proactive approach to diagnostic workup is warranted for patients presenting with relevant neurological symptoms, especially those suggestive of multisystemic manifestations indicative of hEDS.

## 2 The central nervous system

**Figure 1:**
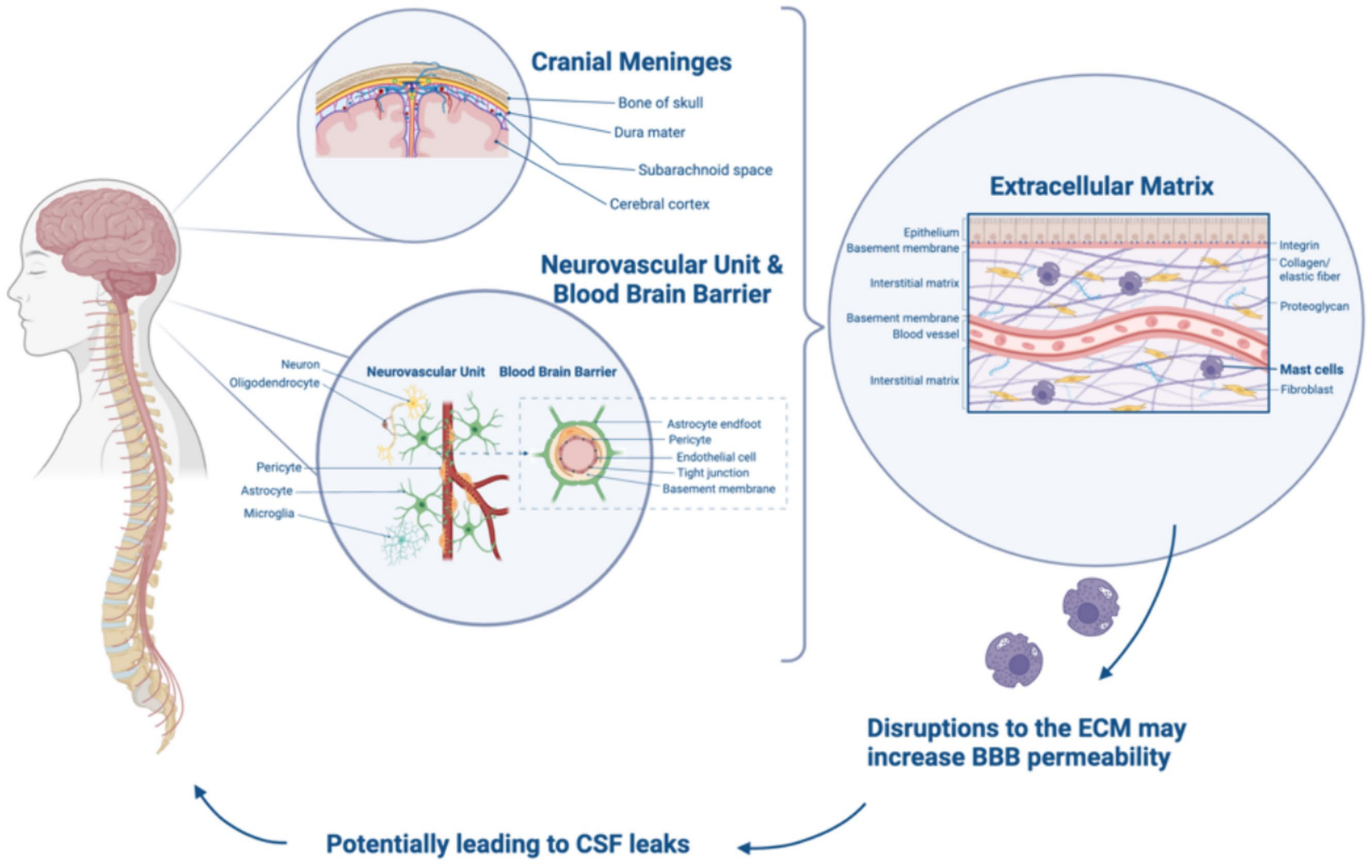
Relationship between cranial meninges, BBB, ECM, and CSF leaks. Created with Biorender.com.

The CNS is responsible for processing and interpreting sensory information from the peripheral nervous system, as well as controlling voluntary and involuntary movements. The CNS is primarily composed of neurons, which allow for the transmission of information throughout the CNS. The delicate structures of the CNS, the brain and spine, are bathed in cerebrospinal fluid (CSF), enclosed by the blood brain barrier (BBB), and protected by bone (1). The BBB, comprised of specialized endothelial cells, pericytes, astrocytes, and extracellular matrix (ECM) components, plays a crucial role as a selective barrier, tightly regulating the passage of substances between the bloodstream and the brain (2). As the BBB serves as a vital defense mechanism for the brain, it consists of three protective layers, the meninges, which play a multifaceted role in brain protection— together they circulate cerebrospinal fluid (CSF), act as a selectively permeable barrier, and provide crucial support to the CNS. The outermost layer is the dura mater, also referred to as the dura, which lies beneath the skull and around the spinal cord. Beneath the dura is a fine, web-like membrane called the arachnoid containing CSF. The innermost layer of the meninges is the pia mater, which is a highly vascularized membrane that adheres to the spinal cord and brain directly, nourishing the underlying neural tissue (3). The meninges are responsible for ensuring CSF can function by acting as a cushion and providing mechanical protection for preventing brain and spinal cord injury and facilitating nutrient transport, waste removal, pressure regulation, immunological functions, and more (Figure 1). The meninges are connected to the perineural sheaths of the cranial and paraspinal motor and sensory nerves making these structures vulnerable to compressive issues if meningeal integrity is compromised. Meningeal abnormalities may lead to conditions such as ectasia, characterized by the dilation or distention of tubular structures, which can result in Tarlov cysts and neuropathies. Additionally, these abnormalities can cause papilledema, which is swelling of the optic disc, in the posterior part of the eye due to increased intracranial pressure (4).

## 3 The extracellular matrix (ECM)

The extracellular matrix (ECM) is a complex network of proteins and carbohydrates that provide structural and biochemical support to the cells present within all tissues. The ECM plays a role in tissue development, maintenance, repair, cell signaling, and adhesion. Key components of the ECM include collagens, elastin, fibronectin, laminin, glycosaminoglycans, proteoglycans, and glycoproteins (2). Fibroblasts, the most common cell type in connective tissue, are crucial for synthesizing the ECM and maintaining its integrity. Dysregulated fibroblast function can contribute to various pathological conditions, including connective tissue disorders (CTDs) (5).

Collagen, the most abundant protein in the ECM, provides structural support to tissues. The human body contains at least 28 types of collagen, each serving specific functions, with types I, II, and III being the most prevalent. In the blood–brain barrier (BBB), the basement membrane (BM) integrates ECM proteins such as collagen and laminin, providing structural support, selective barriers, cell adhesion, and nutrient exchange (3). Collagen IV plays an important role in stabilizing the BM by providing mechanical support to endothelial cells and maintaining the integrity of the BBB (6). Collagen IV has also been detected in cerebral blood vessels of the BM, making up part of the arterial walls and adding both strength and flexibility (4). Receptors like dystroglycan and integrins facilitate cell–cell and cell-matrix interactions within the BBB, regulating signaling pathways (5). This regulation enables cellular adaptations in response to environmental changes. Additionally, these receptors establish a physical connection between the ECM and the cytoskeleton, contributing to the structural integrity of the BBB and anchoring the cells in place. Within the CNS vasculature, fibronectin and collagen IV are secreted by endothelial cells, pericytes, and astrocytes, further contributing to the dynamic cellular environment of the BBB (3). In addition to the BBB, collagen’s role in tendons, ligaments, skin, and cartilage ensures resilience to stress. When CTDs arise, seemingly disparate health issues emerge, impacting joint functionality, wound healing, and organ elasticity. Disruptions or abnormalities in the ECM and collagen are implicated in the development of health conditions including CTDs.

## 4 Hypermobile Ehlers-Danlos syndrome and neurological manifestations

The Ehlers-Danlos syndromes (EDS) are a group of 14 heritable disorders that affect the body’s connective tissue caused by mutations in collagen genes and related components of the ECM (6). Clinical manifestations of EDS include joint hypermobility, skin hyperextensibility, chronic pain, and gastrointestinal, neurological, and immunological comorbidities, requiring multiple specialists for patient care and management (7). There are currently no FDA-approved treatments or curative therapies for EDS, other than addressing symptom management. The most prevalent subtype, hypermobile EDS (hEDS), is more common than previously believed and is the only subtype of EDS that lacks a clearly defined genetic marker (8). The current diagnostic process for hEDS is outlined in the “2017 International Classifications of the Ehlers-Danlos Syndromes” (9). Despite a lack of clear genetic etiology for hEDS, studies have indicated changes in ECM proteins in various tissues from hEDS patients (10–13).

While hEDS occurs on a spectrum, patients may experience neurological symptoms and comorbidities. Neurological symptoms associated with hEDS include fatigue, pain, headache, muscle weakness, and paresthesia with ranging severity. Symptoms can be due to a variety of manifestations and comorbidities including autonomic dysfunction, cerebral spinal fluid (CSF) leaks, Chiari malformation, upper cervical spine instability, changes in intracranial pressure, migraine, and tethered cord syndrome (TCS) (6). The neurological aspects of hEDS can significantly limit one’s ability to complete daily tasks, both physically and cognitively.

In hEDS patients, fatigue is prevalent and often linked to dysautonomia, including postural orthostatic tachycardia syndrome (POTS) (14). While there’s no cure for hEDS-related fatigue, management varies and commonly involves lifestyle changes, physical activity, and medication. Headaches are another common complaint seen in neurological clinics, especially in patients with hEDS. In 1997, a study of 51 individuals with various forms of EDS found that 30–40% of cases reported neck pain and headache (15). An additional study in 2011 reported migraines with or without aura in 75% of joint hypermobility spectrum (JHS) (16). The correlation between connective tissue abnormalities and neurological symptoms remains an ongoing area of research.

## 5 CSF leaks, intracranial hypotension, and intracranial hypertension

**Figure 2:**
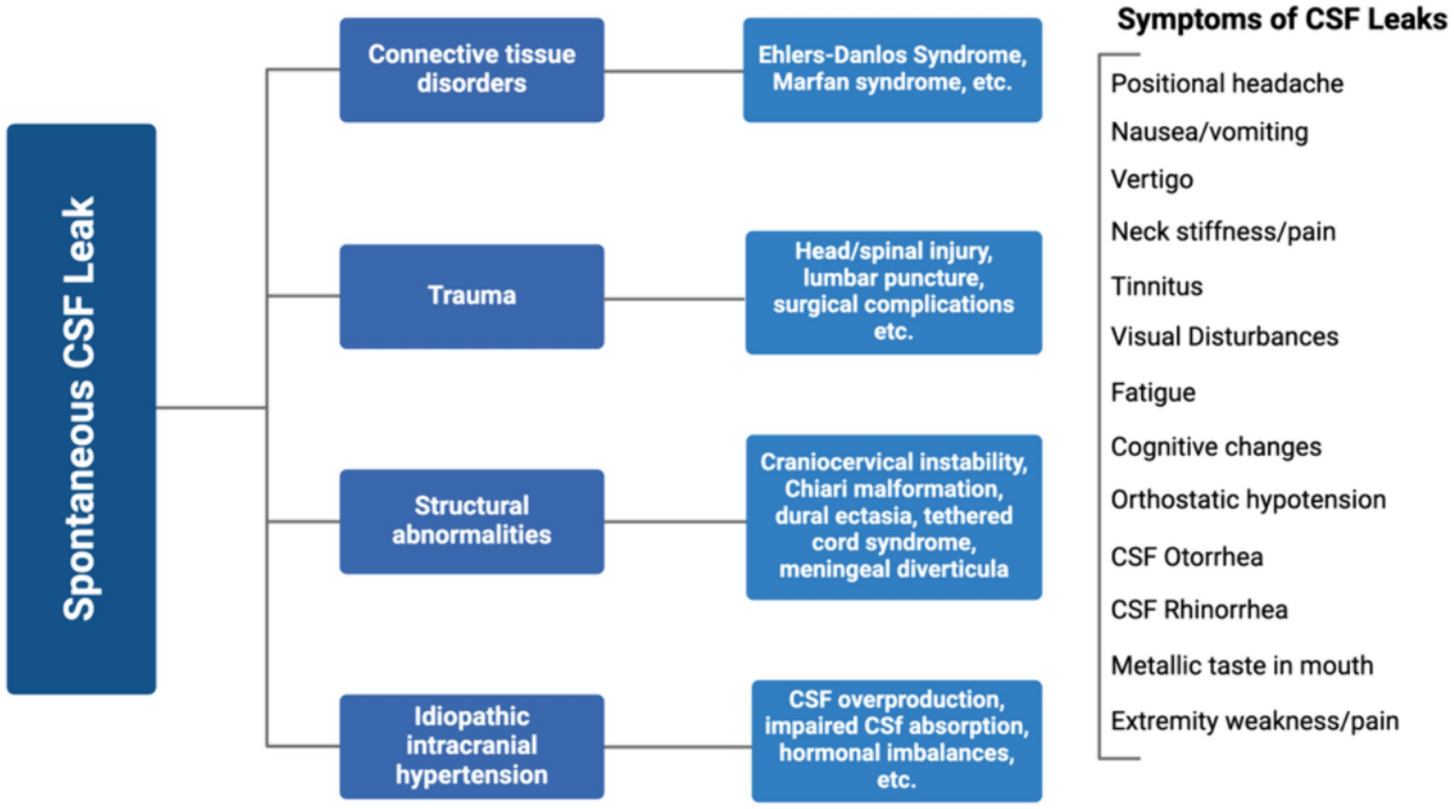
Causes and symptoms of spontaneous CSF leaks. Created with Biorender.com.

Low intracranial pressure, known as intracranial hypotension, results from loss of CSF volume in the subarachnoid space or ectasia, leading to expansion of the thecal sac. Intracranial hypotension is often triggered by a CSF leak which can be caused by dural tears, trauma to the head or spine, lumbar punctures, or less frequently, occur spontaneously (17). The cause of spontaneous CSF leaks may be related to connective tissue disorders, structural abnormalities, idiopathic intracranial hypertension, or trauma injury (Figure 2). Typical MRI findings associated with intracranial hypotension follow the SEEPS mnemonic including “subdural fluid collections, enhancement of the pachymeninges, engorgement of venous structures, pituitary hyperemia, and sagging of the brain” (18, 19). The hydrostatic indifference point, typically in the C7-T1 junction, is where CSF pressure remains constant when moving from erect to recumbent thus intracranial hypotensive findings are less likely to be observed in fistulae anatomically near this area. In a recumbent position, CSF pressure is slightly positive throughout the neuroaxis, and in an erect position, CSF pressure increases below and decreases above this point (20). This explains why skull base CSF leaks usually do not cause orthostatic headaches or display typical imaging features of spontaneous intracranial hypotension. While this is not true for every case, it has become dogmatic in the radiology specialties. Symptoms of CSF leaks include severe positional headaches that worsen when in an upright position, nausea, vomiting, vertigo, neck stiffness, blurred vision, tinnitus (ringing in ears), fatigue, cognitive changes, orthostatic hypotension, metallic taste in the mouth, and extremity weakness/pain (Figure 2) (21). CSF fluid may leak out of the nose (rhinorrhea) or the ears (otorrhea) (Figure 2). While spontaneous intracranial hypotension and CSF leaks aren’t rare, they are considered underdiagnosed (18). In a small study of 11 people presenting with postural headaches (among other neurological complaints), a spontaneous CSF leak was present in all participants (18). Chiari malformation was another frequent finding and underlying CTDs were broadly suspected, if not previously diagnosed.

Intracranial hypertension is characterized by any condition in which there is increased pressure inside the skull. This may be caused by many factors including tumors, infections, meningitis, ischemic stroke, trauma, hydrocephalus, systemic disorders, or congenital malformations (22). Symptoms of intracranial hypertension include headaches, visual disturbances, tinnitus, nausea, and other neurological deficits. Idiopathic Intracranial Hypertension (IIH) involves increased intracranial pressure without a clear cause (23). Diagnosis includes clinical evaluation, imaging, and measuring cerebrospinal fluid pressure via lumbar puncture. Recognizing transverse sinus stenosis as a key factor in intracranial venous hypertension has increased the use of catheter venography to evaluate stenting candidates and advance research into cerebral venous anatomy and disease mechanisms, deepening the understanding of cerebral venous outflow disorders (CVD) within the IIH spectrum. In a retrospective study of 86 patients with diagnosed or suspected CTDs being evaluated for cerebral venous outflow disorders (CVD), the following prevalence of conditions was observed IIH (75.6%), CSF leaks (51.2%), dysautonomia (45.3%), EDS (55.8%), MCAD (25.6%) and systemic venous compression syndrome in (10.5%) (24). This study describes significant differences in patient profile from the typical demographic of obese women of childbearing age from diverse racial backgrounds. The demographic observed in suspected or diagnosed CTDs with IIH have a lower mean BMI and consist mainly of non-obese Caucasian young women. This population presents with more severe symptoms at lower lumbar puncture opening pressures, lower mean superior sagittal sinus pressures on venography compared to standard IIH patients, and report an increased severity of headaches and worse quality of life (24). This suggests a hypersensitivity to pain and/or pressure dysregulation associated with CTDs and highlights the necessity for a better understanding of cerebral venous disorders and connective tissue conditions, as symptoms in these patients may present with increased severity and persist at lower intracranial pressures than typically expected.

Meningeal diverticula, also known as arachnoid diverticula, are an abnormal structure of thin-walled sacs that form within the layers of the meninges. They can be congenital or acquired later in life. The cysts present due to meningeal diverticula cysts are different than syringomyelia cysts as they are typically connected to the subarachnoid space and can accumulate CSF and grow in size. There is some conflicting research concerning the relationship between meningeal diverticula, CSF leaks, and intracranial hypertension, including an observational study which found an elevated percentage of meningeal diverticula in CSF patients and identified these diverticula as a type of CSF leak (25). However, another study noted no difference in the prevalence of diverticula between patients with spontaneous intracranial hypotension and controls, highlighting the need for further investigation into this relationship (26).

hEDS can cause the connective tissue of the dura mater to weaken and thin, leading to a condition known as dural ectasia, where the dura mater bulges outward around the spinal cord (27). This condition is associated with symptoms such as headaches, weakness, low back pain, and numbness, which are often relieved when lying down. hEDS patients may experience additional structural spine issues that can add further stress to the dura mater. These structural issues include Chiari malformation (CM) and craniocervical instability (CCI). Although CM and CCI are not direct causes of CSF leaks, they can cause irregular CSF flow and alter intracranial pressure (28). A study showed that CM models had significantly higher peak CSF pressure compared to healthy controls.

Syringomyelia, often associated with CM, is also associated with hEDS, but is characterized by the development of a fluid-filled cyst (syrinx) within the spinal cord (27). This can expand over time and damage nerve fibers, causing pain, loss of sensation, temperature dysregulation, impaired reflexes, and muscle atrophy. A 2013 study comparing 10 healthy volunteers and 18 patients with CM, observed significantly faster and earlier caudal CSF flow in CM patients. However, no significant differences in CSF velocities were noted between healthy volunteers and patients with syringomyelia in the study (29).

Meningiomas, benign tumors from the meningeal layers of the brain or spinal cord, can exert pressure on the dura mater, potentially causing tears and CSF leaks (30). While meningiomas can contribute to CSF leaks, they are rarely the primary cause. The relationship between meningiomas and hEDS is poorly understood and requires further research.

Vascular insufficiency in CTDs can occur in the cranial, cervical, or systemic central venous regions of the thorax, thoracic outlet, abdomen, and pelvis, potentially leading to abnormalities in CSF pressure and breakdown of the BBB through spontaneous fistulae (31). Patients with CTDs are thought to have an elevated risk for several cerebrovascular conditions such as intracranial aneurysms, arterial dissections, and acute ischemic strokes (32). This increased risk is primarily attributed to genetic mutations affecting collagen and proteoglycans in the ECM, which weaken the walls of blood vessels. It is important that neurovascular specialists be included in the care of patients with CTDs and potential cerebrovascular complications. Additionally, a strong correlation of EDS seen in patients with three of more vascular compression syndromes, as observed in a study of over 250 patients with vascular compressions syndromes (33). The relationship between EDS, vascular compressions, vascular insufficiency, and CSF leaks is poorly understood and an area where further research would be beneficial.

In a retrospective study of 86 patients with diagnosed CTDs and cerebral venous outflow disorders including idiopathic intracranial hypertension (IIH), significant comorbidities were identified including POTS (55.8%), dysautonomia (45.3%), CCI (37.2%), MCAD (25.6%), and TCS (23.3%), while a second study of 2,149 patients with hEDS found 70.87% has dysautonomia/POTS, 31.60% had CCI/AAI, 7.91% had CM, and 6.75% had TCS (24, 34). Some cases of CSF leaks may be linked to TCS or tethered cord release surgeries, considering the possibility of spinal cord tension impacting the flow of CSF (35).

## 6 MCAD and CSF leaks

The autonomic nervous system (ANS) regulates essential functions like heart rate, blood pressure, digestion, and vessel tone through specific anatomic loci and includes the sympathetic and parasympathetic branches. The sympathetic nervous system (SNS) originates in the thoracolumbar spinal cord and affects organs through the sympathetic chain ganglia. The parasympathetic nervous system (PNS) arises from the craniosacral regions, with fibers in cranial nerves III, VII, IX, and notably the vagus nerve (X), which provides is essential to maintaining homeostasis and the regulation of involuntary functions. This division allows the SNS to handle stress while the PNS supports rest and digestion (36, 37). These regions highlight the intersection of anatomy and physiology, where structural dysautonomia and mast cell dysfunction may arise. Understanding the anatomy of the ANS is vital to properly managing non-structural, non-surgical patients with hEDS, autonomic dysfunction, and MCAD.

Patients with hEDS have an increased incidence of MCAD, where mast cells overreact to stimuli, causing symptoms such as allergic reactions, skin sensitivity, itching, temperature dysregulation, gastrointestinal issues, and anaphylaxis (34). A recent review indicates that nearly 1 in 3 patients with MCAD had a comorbid diagnosis of hEDS in a sample size of 37,665 patients (38). Mast cells are found in the CNS and are capable of migrating across the BBB when the barrier is compromised due to CNS pathology. Nearly 97% of mast cells are found on the brain side of blood vessels and can communicate with the ECM, neurons, astrocytes, and blood vessels (39). They release inflammatory mediators such as histamine, proteases, and cytokines, playing a key role in the body’s defense mechanisms (40). Abnormal mast cell activation disrupts connective tissue integrity through the actions of mediators including histamine and tryptase, impacting multiple organ systems and leading to MCAD (38).

Histamine, a potent vasodilator, increases blood vessel permeability, potentially heightening the blood–brain barrier (BBB) permeability and allowing normally restricted substances to enter the brain (41, 42). Tryptase is a serine protease stored in the granules of mast cells that can degrade ECM proteins, promote inflammation by stimulating the release of cytokines, and is commonly used as a measure of mast cell degranulation (42). One study of cuprizone administration showed changes in tight junction protein expression and an increase in mast cell presence and tryptase expression in the cortex and corpus callosum, indicating their activation and potential role in inducing changes in the permeability of the BBB (43). Chymase, another serine protease, can degrade extracellular matrix components and contribute to inflammatory processes. Further evidence of the interaction between mast cell activation and the BBB includes a study on the Japanese encephalitis virus (JEV) that reported chymase inhibition reversed BBB leakage in mice, therefore suggesting chymase as a factor influencing the permeability of the BBB (44).

Mast cell stabilizers block the mast cells’ effects on vascular permeability as shown in W/Wv mice. Furthermore, several reports state that BBB permeability is regulated by brain histamine and serine proteases which cause small blood vessels to leak and lead to neuronal hyperexcitability and inflammation through activating certain receptors (39). Additional evidence, from a study on compound 48/80 in doves, demonstrated that mast cell degranulation increased BBB permeability, suggesting that mast cell activation can alter BBB integrity locally (45). This increased leakiness can directly impact the progression of neurologic disease (2). Connective tissue abnormalities may contribute to increased BBB leakiness, leading to brain inflammation if immune cells and cytokines penetrate brain tissue (46). Such permeability issues in the BBB due to hEDS and mast cell degranulation may compromise the barrier’s integrity, leading to CSF leaks.

## 7 Discussion

The BBB and CNS are composed of and supported by ECM components, which can be affected in CTDs such as hEDS. Disruptions in ECM composition can impair the BBB’s structural and functional integrity, contributing to the higher prevalence of CSF leaks in hEDS patients compared to the general population. These patients often endure symptoms including positional headaches, neck stiffness, blurred vision, tinnitus, lightheadedness, and cranial nerve dysfunction. Given the underdiagnosis of both hEDS and CSF leaks, it is crucial to consider a diagnostic workup for CSF leaks in patients presenting with relevant symptoms, especially if they exhibit multisystemic manifestations suggestive of hEDS. Further research into the relationship between CSF leaks, intracranial hypotension, intracranial hypertension, and neurological issues common in hEDS patients, such as craniocervical instability (CCI), Chiari malformation (CM), systemic vascular compression syndromes, tethered cord syndrome, and syringomyelia, could shed light on why CSF leaks often accompany these disorders.

Additionally, mast cell activation disorder (MCAD) may contribute to spontaneous CSF leaks in hEDS patients, suggesting that managing mast cell activity could be a less invasive way to prevent recurrent CSF leaks. Particularly, analyzing specific mast cell mediators such as histamine, tryptase, and chymase. Histamine can increase vascular permeability meanwhile tryptase and chymase can degrade ECM components such as fibronectin and collagen, further weakening the connective tissue.

Understanding the interplay between ECM disruption due to hEDS and the activity of mast cell mediators will provide insight into potential therapeutic interventions. Perhaps, targeting specific mast cell mediators and stabilizing their activity could strengthen connective tissue and reduce the incidence of spontaneous CSF leaks among hEDS patients. A study on sepsis-associated encephalopathy demonstrates that mast cell activation weakens the BBB in sepsis and that early treatment with the mast cell stabilizer, cromolyn, assists by reducing the chain reaction of inflammation (47). Perhaps, similar uses of mast-cell stabilizers such as cromolyn could assist in reducing the cascade of neuroinflammation and ultimately reduce CSF leaks in patients with hEDS and MCAD.

## Author contributions

SS: Conceptualization, Investigation, Writing – original draft, Writing – review & editing. VD: Conceptualization, Investigation, Writing – original draft, Writing – review & editing. TP: Conceptualization, Investigation, Writing – original draft, Writing – review & editing. CG: Conceptualization, Investigation, Writing – original draft, Writing – review & editing. SP: Conceptualization, Investigation, Writing – original draft, Writing – review & editing. RN: Writing – original draft, Writing – review & editing.
